# Hearing and dementia

**DOI:** 10.1007/s00415-016-8208-y

**Published:** 2016-07-02

**Authors:** Chris J. D. Hardy, Charles R. Marshall, Hannah L. Golden, Camilla N. Clark, Catherine J. Mummery, Timothy D. Griffiths, Doris-Eva Bamiou, Jason D. Warren

**Affiliations:** 1 Department of Neurodegenerative Disease, Dementia Research Centre, UCL Institute of Neurology, University College London, Queen Square, London, WC1N 3BG UK; 2 Department of Neuro-otology, National Hospital for Neurology and Neurosurgery, Queen Square, London, UK; 3 UCL Ear Institute, University College London, London, UK; 4 Cognitive Disorders Clinic for the Deaf, National Hospital for Neurology and Neurosurgery, Queen Square, London, UK; 5 Auditory Group, Institute of Neuroscience, The Medical School, University of Newcastle upon Tyne, Newcastle upon Tyne, UK; 6 Central Auditory Disorders Clinic, National Hospital for Neurology and Neurosurgery, Queen Square, London, UK

**Keywords:** Hearing, Auditory, Dementia, Alzheimer’s disease, Frontotemporal dementia, Progressive aphasia, Lewy body disease

## Abstract

Hearing deficits associated with cognitive impairment have attracted much recent interest, motivated by emerging evidence that impaired hearing is a risk factor for cognitive decline. However, dementia and hearing impairment present immense challenges in their own right, and their intersection in the auditory brain remains poorly understood and difficult to assess. Here, we outline a clinically oriented, symptom-based approach to the assessment of hearing in dementias, informed by recent progress in the clinical auditory neuroscience of these diseases. We consider the significance and interpretation of hearing loss and symptoms that point to a disorder of auditory cognition in patients with dementia. We identify key auditory characteristics of some important dementias and conclude with a bedside approach to assessing and managing auditory dysfunction in dementia.

## Introduction

Although hearing impairment is not generally regarded as a cardinal feature of dementia, hearing in patients with dementia is a focus of growing clinical interest. Recent evidence suggests that hearing loss may predict or accelerate cognitive deterioration [1–3], and alterations of hearing may manifest as complex cognitive and behavioural symptoms relevant to the differential diagnosis of dementias [4–10]. Interventions targeting auditory processes (most notably, music) have gained wide currency [4, 11]. However, the organisation of the human auditory brain is complex and incompletely understood. Moreover, neuropsychological frameworks for characterising hearing disorders produced by brain disease and practical tools for assessing auditory functions suitable for use in cognitively impaired patients are often lacking.

In this review, we outline a clinically oriented, symptom-based approach to hearing in dementia, informed by recent progress in the clinical auditory neuroscience of these diseases. We consider the problem of hearing loss (impaired detection of sound and how this interacts with cognitive function) and symptoms that point to a disorder of auditory cognition (impaired understanding or behavioural responses to sound). We identify key auditory characteristics of some important dementias. We conclude with a bedside approach to assessing and managing auditory dysfunction in dementia.

## The auditory brain and dementia

### Neuropsychology of hearing

Hearing (considered broadly as the function of the human auditory brain and its peripheral end organs) has been aligned with other complex neuropsychological processes based on studies of the normal brain and focal brain damage [12, 13]. Together, this evidence suggests a hierarchical organisation that differentiates categories and stages of auditory information processing (Table 1). Processing of sound begins in the ascending auditory pathways extending from cochlea to primary auditory cortex in Heschl’s gyrus: this is not a passive relay but involves considerable signal transformation [12]. While the terminology of hearing disorders is problematic, in consideration of disease associations, it is useful to attempt to distinguish between peripheral (predominantly cochlear or auditory nerve), subcortical (ascending auditory pathway), and cortical auditory dysfunction. Auditory cognition—processing beyond sound detection leading to auditory perception and understanding—is mediated by distributed networks involving auditory cortex and its cerebral connections; disorders affecting these networks produce characteristic symptoms and syndromes of auditory cognitive dysfunction (summarised in Table 1).

**Table 1:** An outline of core operations in auditory cognition and their clinical and neuroanatomical correlates

Auditory cognitive operation	Clinical correlates	Neuropsychological tests	Procedure^a^	Neuroanatomical correlates [13, 82]
Feature detection	Cortical deafness^b^, tinnitus^c^	Sound detection Gap-in-noise detection AM/FM^d^ detection Spatial lateralisation	Detection of any sound (e.g., tone) behaviourally/EP [83] Detection of short silent interval in white noise burst [76] Detection of modulation (vibrato) of intensity/pitch in carrier tone [76] Detection of right-left sound shift based on inter-aural phase/intensity cues [76]	PAC, lat HG, PT, pSTG, subcortical circuits
Feature analysis	Word deafness^e^, dystimbria^f^, amusia^g^	Phoneme discrimination MBEA pitch/temporal subtests	Discrimination of sound pairs/sequences differing in pitch, temporal or timbral characteristics [49, 79, 84] Labelling of features in a single sound (e.g., tone glide direction ‘up’ or ‘down’) [7]	lat HG, pSTG/STS, aSTG, subcortical circuits
Scene analysis	Auditory disorientation	SSI-ICM Speech-in-noise^h^ Spatial localisation Dichotic listening	Identification of a sentence spoken over background message same ear [34] Identification of words against background noise/multi-talker babble Discrimination of sound location/movement in real or virtual space [9, 10] Attention to one of two stimuli played simultaneously via each ear [23]	PT/pSTG, IPL, PFC, hippocampus, subcortical circuits^i^
Object representation (apperceptive processing)	Auditory apperceptive agnosias, musical and verbal hallucinations	Melody discrimination Distorted melodies Voice discrimination Accent processing	Discrimination of (unfamiliar) melodies [56, 85] Identification of an altered familiar melody [85] Discrimination of (unfamiliar) speakers [57] Speech perception under unfamiliar accents [8, 52]	PT, pSTG/STS, IPL, aSTG
Object recognition (semantic processing)	Auditory associative agnosias (including phonagnosia)	Environmental sound, melody, voice recognition	Recognition of familiar sounds, tunes, voices; conventionally assessed by naming the target but can be assessed by forced-choice or matching cross-modally (e.g., sound–picture) [56] or within-modality (perceptually dissimilar sound excerpts, categorisation based on semantic characteristic) [7, 49, 54, 55], familiarity decision [56] in patients with aphasia	aSTG, TP, insula
Emotional valuation	Receptive dysprosodia, auditory anhedonia, Musicophilia	Emotion recognition Emotional response	Naming, forced choice [55, 64, 66, 86] or cross-modal labelling of emotions in sounds Behavioural rating of valence, arousal; autonomic indices [68]	MTL, insula, OFC, ACC, mesolimbic/striatal circuits
Working memory/attention^j^	Auditory neglect/inattention	Compare sequential sounds Oddball detection Dichotic listening	*n*-back tasks (e.g., [77]) Sustained attention with detection of target deviants behaviourally/EP [87] Attention to one of two stimuli played simultaneously via each ear [75]	Fronto–parieto–temporal, subcortical circuits

*ACC* anterior cingulate cortex, *AM/FM* amplitude/frequency modulation, *a/pSTG* anterior/posterior superior temporal gyrus, *EP* electrophysiological evoked potentials, *IPL* inferior parietal lobe, *lat HG* lateral Heschl’s gyrus, *MBEA* Montreal Battery for Evaluation of Amusia, *MTL* mesial temporal lobe, *OFC* orbitofrontal cortex, *PAC* primary auditory cortex, *PFC* prefrontal cortex, *PT* planum temporale, *SSI-ICM* synthetic sentence identification with ipsilateral competing message, *STS* superior temporal sulcus, *TP* temporal pole

^a^Few widely available tests or population norms are available for auditory cognition. These are mainly used in research settings, but certain instruments may be suitable for systematic clinical assessment of cognitively impaired patients (e.g., Newcastle Auditory Battery [76]; Montreal Battery for Evaluation of Amusia [77]; Queen Square Tests of Auditory Cognition for auditory object processing, voice and scene analysis [6–9, 48, 57])

^b^Subtotal cortical deafness often manifests as auditory agnosia

^c^Tinnitus is mediated by distributed circuitry also including subcortical, anterior and limbic structures

^d^Neuroanatomical correlates vary with modulation rate

^e^Mechanism of word deafness may be heterogeneous

^f^Impaired perception of timbre (that property distinguishing two sounds of identical pitch, duration and loudness, e.g., musical instrument voices)

^g^Impaired perception of music due to a cerebral cause

^h^Speech-in-noise perception is impaired with cochlear dysfunction so interpretation of any more central deficit must be cautious [23]

^i^Maintaining alertness and attention

^j^Particularly during auditory scene analysis but relevant to auditory sequence processing more generally (auditory neglect/inattention unusual in dementia but impaired monitoring of acoustic events common)

As a framework for analysing disorders of auditory cognition, it is useful to consider complex sounds (speech, voices, music, and environmental noises) as ‘auditory objects’ that must be disambiguated from the auditory background and organised into coherent perceptual representations [13]. The processing of such sound objects entails perceptual analysis (encoding of acoustic features, such as pitch, rhythm, and timbre) leading to semantic processing (extraction of associated meaning, leading to sound recognition) [13, 14]. In the world at large, sounds are embedded in auditory scenes that must be actively deconstructed to identify and track sounds of interest [10]: this, in turn, requires the representation of sound location and movement (auditory spatial analysis) and abstraction of identifying sound characteristics under varying listening conditions (auditory apperceptive processing). Many sounds also have emotional and behavioural relevance.

### The burden of dementia

Dementia is arguably the most significant public health problem confronting ageing societies, with an estimated 800,000 sufferers currently in the United Kingdom alone [15]. However, ‘dementia’ designates a syndrome of acquired, progressive, socially and/or occupationally significant cognitive and/or behavioural decline: this definition embraces over a hundred highly diverse diseases, the most common of which is Alzheimer’s disease (AD) [4, 16–18]. Here, we focus on major neurodegenerative causes of dementia in mid to later life, collectively characterised by pathogenic protein spread over large-scale cerebral networks and distinctive profiles of regional brain atrophy and clinical deficits (summarised in Table 2). Brain networks targeted by these diseases overlap the temporal, parietal, frontal, and subcortical circuitry that supports auditory cognition (Tables 1, 2): this is key to anticipating and understanding the disorders of hearing that accompany particular dementia syndromes.

**Table 2:** Summary of major neurodegenerative dementias, emphasising auditory characteristics

Disease/syndrome	Core clinical phenotype	Key auditory symptoms	Auditory cognitive processes^a^					Pathological neuroanatomy^b^
			Perc	App	Sem	Em	Wm/Att	
AD: typical [6–10, 57, 64, 88–94]	Episodic, topographical memory loss, parietal deficits	Difficulty tracking sound objects and information in busy acoustic environments, auditory disorientation, increased sound sensitivity	**+**	**++**	**+**	−	**++**	PCC, precuneus, temporo-parietal cortices
*PCA* ^c^ [9]	Visuo-perceptual, visuo-spatial, other parietal deficits		**+**	**+**				
*LPA* ^c^ [7, 48, 50, 64]	Anomia, phonemic and verbal working memory deficits		−	**+**	**+**	**+**	**++**	
PDD/DLB^d^ [59, 60, 87, 95]	Fluctuating executive, attentional deficits, bradyphrenia, visual hallucinations, parkinsonism	Auditory hallucinations	**+**			**+**	**+**	Cortico–subcortical circuits
FTLD: sporadic/undefined								
*bvFTD* [51, 54, 68, 96, 97]	Socio-emotional, executive dysfunction with disinhibition, apathy, obsessionality, other behavioural abnormalities	Sound aversion, phonagnosia, altered attentive processing of auditory stimuli	−	−	**+**	**++**	**++**	Auditory and multimodal association cortex in ant TL, OFC, insula, ACC, striatal circuits
*SD* [5, 48, 49, 51, 53, 54, 56, 57, 68, 98]	Vocabulary loss, visual agnosia due to impaired semantic memory, behavioural changes similar to bvFTD	Musicophilia, tinnitus; phonagnosia/nonverbal sound agnosia	−	**+**	**++**	**++**	−	Auditory/multimodal association cortex in ant TL, OFC, insula
*PNFA* [7, 8, 48, 49, 52, 64]	Speech production deficits, agrammatism	Agnosia for environmental sounds, accents, word deafness	**++**	**+**	**+**	**+**	**++**	Peri-Sylvian networks
FTLD: genetic								
*MAPT* [51, 54, 99]	Similar bvFTD, may have associated parkinsonism	Altered hedonic responses to sound	**+**		**++**	**++**		Ant TL/fronto–subcortical network
*C9orf72* [56, 68, 99, 100]	Similar bvFTD or PNFA, may have associated motor neuron features	Auditory hallucinations	−		**+**	**+**	**+**	Cortico–thalamo–cerebellar network
*GRN* ^e^ [7, 64, 101, 102]	Similar bvFTD or mixed aphasia, often prominent parietal signs	Limited information	**+**	**+**	−	−	**+**	Distributed intra-hemispheric networks
CBS/PSP [103, 104]	Executive deficits, bradyphrenia in context parkinsonism, supranuclear gaze palsy, limb dystonia – apraxia	Agnosia for environmental sounds, disordered voice emotion processing	−		**+**	**+**		Cortico–subcortical circuits, IFG
HD^d^ [105, 106]	Executive and behavioural changes with chorea	Attentive processing of auditory stimuli	**+**				**+**	Cortico–subcortical circuits
Prion diseases [40, 44, 67]	Usually rapid global dementia with prominent myoclonus, ataxia; wide phenotypic variation (especially genetic forms)	Occasionally tinnitus, cortical deafness, auditory hallucinations, increased sound sensitivity	**+**	**+**				Primary auditory cortex

*ACC* anterior cingulate cortex, *AD* Alzheimer’s disease, *ant TL* anterior temporal lobe, *App* auditory apperception (including parsing of auditory scenes into constituent sound objects), *bvFTD* behavioural variant frontotemporal dementia, *C9orf72* mutations in open reading frame 72 on chromosome 9, *CBS/PSP* corticobasal syndrome/progressive supranuclear palsy, *Ep mem* episodic memory for nonverbal sounds (including music), *Em* emotion processing from sounds (including music/prosody), *FTLD* frontotemporal lobar degeneration, *GRN* progranulin gene mutations, *HD* Huntington’s disease, *LPA* logopenic aphasia, *MAPT* microtubule-associated protein tau gene mutations, *OFC* orbitofrontal cortex, *PCA* posterior cortical atrophy, *PCC* posterior cingulate cortex, *Perc* early auditory perception (acoustic feature detection and analysis), *PDD/DLB* Parkinson's disease/Lewy body dementia, *PNFA* progressive nonfluent aphasia, *SD* semantic dementia, *Sem* semantic processing of sounds (including melodies), *Wm/Att* nonverbal auditory working memory/attention

+ deficit documented, ++ particularly severe in relation to other deficits, − deficit absent/inconsistent, blank cells indicate no adequate data available

^a^Defined by performance on behavioural tests

^b^Distribution of pathological changes in brain networks relevant to auditory deficits, as assessed using voxel-based morphometry, functional MRI and/or post mortem material

^c^Underpinned by Alzheimer pathology in >80 % of cases

^d^Abnormalities of rhythm processing in basal ganglia and cerebellar degenerations [95, 107]

^e^Limited information currently for progressive aphasia presentation only

## Hearing loss and dementia

### Epidemiological evidence

Significant hearing loss (operationally, >20 dB elevation of threshold for pure tone detection) affects around 40 % of those aged over 65 [19] and has important links to cognitive impairment and dementia. Age-related hearing loss (presbycusis) commonly results from cochlear dysfunction, though age-related alterations in more central auditory pathways may also be relevant and have probably been under-recognised [20]. The balance of epidemiological evidence across populations suggests that hearing loss is associated with cognitive decline and constitutes a risk factor for development of dementia in older adults, though the strength of this association is somewhat variable [20, 21]. One meta-analysis concluded that cognitive and hearing impairment are correlated and that hearing loss impacts on multiple domains of cognition [21]; this is not simply attributable to hearing loss confounding speech-based cognitive tasks [20] and has been observed in those with and without dementia [22]. Hearing loss ~25 dB has an effect on cognitive deterioration equivalent to around 7 years of ageing [1] and risk of dementia increases with increasing severity of hearing impairment [2].

### The role of peripheral hearing

While the association between hearing loss and cognitive decline appears robust, the mechanism remains unresolved. Hearing impairment might accelerate cognitive decline by compounding sensory and social isolation, increasing cognitive load, and thereby exhausting compensatory cognitive reallocation, or constitute a nonspecific marker of frailty [20]. However, the association between impaired hearing and cognition remains after controlling for other demographic and comorbidity factors [2, 23]. Peripheral hearing loss might hasten neurodegenerative processes more directly. Hearing loss in older adults correlates with tissue volume loss in auditory cortex [24], temporal lobe, and whole brain [3], and is associated with functional reorganisation of auditory cortical networks consistent with more effortful listening and reduced cognitive reserve [25]. Though limited histopathological evidence is available concerning the auditory system in common dementias, major auditory relay nuclei are involved pathologically in AD [26, 27], while animal models suggest that peripheral deafferentation disrupts hippocampal function [28, 29].

### The role of ‘central’ auditory processing

Auditory deficits in AD may be disproportionate to any abnormality of sound detection or otological markers [20, 30–34]: while the neuroanatomical correlates of ‘central’ hearing measures have not been fully defined, such deficits may reflect disordered cortical mechanisms of auditory scene analysis (Table 1). This is corroborated by other evidence that abnormalities of auditory cortical evoked potentials predate clinical symptoms in young carriers of pathogenic AD mutations [35]. Information for other dementias remains very limited. Relatively, a few studies of hearing in dementia have addressed cortical auditory processing specifically, perhaps partly accounting for the wide variation in reported frequency of hearing impairment in AD [4, 36]: an observation that seems otherwise difficult to reconcile with epidemiological data.

The effects of hearing impairment on cognitive decline might be most parsimoniously considered as an interaction of peripheral and more central factors. The auditory system has extensive efferent as well as afferent traffic [12] allowing for reciprocal interaction between cortical, brainstem, and peripheral mechanisms [37]. Moreover, in practice, these can be challenging to disambiguate in individual patients.

### Syndromes of dementia and hearing loss

Syndromic associations of dementia with dysfunction of cochlea or ascending auditory pathways are uncommon and generally occur in the context of more complex neurological impairment, often in younger patients; examples are summarised in Table 3.

**Table 3:** Some syndromes with peripheral or subcortical hearing impairment and dementia

Disease	Aud	Cogn	Associated features	Diagnostic investigations
Inflammatory				
Antiphospholipid syndrome [108]	F; C, RC^a^	F	Headache, seizures, chorea, myelopathy, optic neuritis, vestibulopathy	Antibody profile with compatible clinical phenotype
Multiple sclerosis [109]	U; RC^a^	F^b^	Diverse: vertigo, optic neuritis, various brainstem, cerebral, spinal signs	Compatible clinical and MRI features of CNS demyelination, (McDonald criteria), supported by CSF unmatched oligoclonal bands
Neuro-Behçet’s [110]	F; RC^a^	U^c^	Vestibulopathy, uveitis, headache, brainstem signs, hemiparesis, cerebral venous thrombosis; oral/genital ulcers	None specifically; International Study Group criteria (with pathergy test) for systemic disease
Neurosarcoidosis [111]	U; RC^a^	F	Vestibulopathy, cranial nerve palsies, seizures, aseptic meningitis, myelopathy, peripheral neuropathy, pituitary dysfunction	Contrast MRI sensitive but not specific; whole body PET, biopsy involved peripheral tissue
Susac’s syndrome [112]	T; C^a,d^	T	Retinal artery occlusions; migraine, ataxia, vertigo, long tract signs	MRI (callosal ‘snowball’ lesions); retinal fluoroscein angiography (multifocal distal arteriolar occlusions)
Infectious				
Cryptococcal meningitis [113]	U; RC^a^	F	Headache, papilloedema, seizures, vestibulopathy, cranial nerve palsies; more common in immunocompromised patients	CSF Cryptococcal antigen
Neuroborreliosis [114]	U; RC^e^	U^f^	Lymphocytic meningitis with cranial palsies, vestibulopathy	Lyme serology
Neurosyphilis [115]	U; RC^a,g^	T	Chorioretinitis, Argyll Robertson pupils, vestibulopathy, cranial nerve palsies and brainstem signs, myelopathy (tabes dorsalis), brain infarcts	Treponemal serology (blood and CSF)
Genetic				
CADASIL [116]	U; C^a^	T	Migraine, stroke, psychiatric disturbance	Characteristic MRI with marked anterior temporal/external capsule white matter involvement *Notch3* mutations
MELAS/other mitochondrial syndromes [117]	T/F; C	T/F	Migraine, seizures, stroke-like episodes, ophthalmoplegia, myopathy, lactic acidosis, diabetes mellitus	Various mitochondrial DNA mutations
HSAN IE [118]	T; C, RC?^h^	T	Sensory and autonomic neuropathy, optic neuropathy, narcolepsy	*DNMT1* mutations
IBMPFD [119]	U; RC?	T	Frontotemporal dementia with inclusion body myositis, Paget’s disease of bone	*VCP* mutations
Niemann-Pick type C [120]	F; RC	T^c^	Ataxia, supranuclear gaze palsy, dystonia, psychiatric features, cataplexy, seizures, splenomegaly	Skin fibroblast studies (accumulation of unesterified cholesterol), genotyping
Oculo-leptomeningeal amyloidosis [121]	F; RC?^a^	F	Seizures, stroke-like episodes, headache, ataxia, myelo-radiculopathy, subarachnoid haemorrhage, ocular amyloid	Abnormal meningeal enhancement on contrast MRI Transthyretin mutations
Refsum disease [122]	F; RC	U	Retinitis pigmentosa, anosmia, polyneuropathy	Raised plasma phytanic acid *PHYH* mutation
Spinocerebellar ataxias: [83]	F; C, RC^i^	F	Truncal/limb ataxia, bulbar deficits, proprioceptive impairment, neuropathy, variably prominent across group	Various mutations (most frequently, trinucleotide repeat expansions)
Friedreich’s ataxia [123]			Cardiomyopathy, diabetes mellitus (adult onset milder)	*FXN* expansions
SCA13 [124]			Gait/limb ataxia, dysarthria, hyperreflexia, vibration sense loss	*KCNC3* mutations
Wolfram’s syndrome [125]	T; RC	F^j^	Optic atrophy, diabetes	*WFS1* mutations
Other				
Prion diseases [126]	U; RC	T	Rapid neurological decline, often with prominent myoclonus and ataxia	Increased cortical/basal ganglia signal on DWI/FLAIR MRI with compatible clinical phenotype; rarely prion gene mutation (*E200K*)
Superficial siderosis [127]	T; RC^k^	F	Cerebellar ataxia, pyramidal signs, bladder dysfunction, anosmia, anisocoria; may have history compatible with chronic subarachnoid bleeding	Haemosiderin rimming brain/spinal cord on susceptibility-weighted MRI

*CADASIL* cerebral autosomal dominant arteriopathy with subcortical infarcts and leukoencephalopathy, *CJD* Creutzfeldt-Jakob disease, *DNMT1* DNA cytosine-5-methyltransferase 1 gene, *DWI/FLAIR* diffusion weighted/fluid-attenuated inversion recovery sequences, *FXN* frataxin gene, *HSAN IE* hereditary sensory and autonomic neuropathy with dementia and hearing loss type IE, *IBMPFD* inclusion body myositis with Paget’s disease of bone and frontotemporal dementia, *KCNC3* potassium channel Kv3.3 gene, *MELAS* mitochondrial encephalopathy with lactic acidosis and stroke-like episodes, *PHYH* phytanoyl-CoA 2-hydroxylase gene, *VCP* valosin containing protein gene, *WFS1* wolframin gene

The Table excludes paediatric disorders that do not also present during adult life; auditory (Aud) and cognitive (Cogn) phenotypes have been classified according to whether clinical impairments of hearing and/or cognition are: *T* typical of the entity (a very frequent or defining feature), *F* frequent (a common association), *U* unusual (a recognised association). The cognitive phenotype in most cases is not diagnostic, comprising variably prominent executive, subcortical and behavioural deficits and affective changes. The auditory phenotype has been classified according to the origin of hearing loss, where (often limited) information available: *C* cochlear, *RC* retrocochlear (auditory nerve and/or brainstem pathways)

^a^May be sudden

^b^Generally more significant in progressive forms

^c^May have prominent neuropsychiatric changes

^d^Low-to-mid-frequency loss characteristic

^e^May have persistent post-treatment altered hearing (e.g., loudness intolerance)

^f^Subjective cognitive symptoms frequent

^g^Ménière’s-like presentations may occur

^h^Mid-frequency loss

^i^Auditory brainstem pathway involvement appears more functionally significant than more peripheral involvement and may disrupt temporal processing leading to deficits of spatial and speech perception (frequency of impairment varies with mutation)

^j^May be more prominent in later disease

^k^Primary cochlear damage may also contribute

## Symptoms of altered auditory cognition in dementia

Though auditory dysfunction is rarely the presenting feature, histopathological involvement of auditory cortices has been described in major neurodegenerative dementias [26, 38–41], and deficits of auditory cognition (Table 1) are not uncommon early features of these diseases. Certain general observations suggest an auditory cognitive disorder: the patient typically experiences greater listening difficulties and derives less benefit with conventional binaural amplification than anticipated from the degree of audiometric loss and may also exhibit various abnormal behavioural responses to sounds. Matching of incoming sound information to stored neural ‘templates’ based on past experience of the auditory world may be a general operating principle of the auditory brain [14, 42]: disruption of this process with neurodegenerative pathologies may lead to deficient perception or to aberrant perception of sounds. Deficient sound perception or recognition not attributable to faulty peripheral encoding constitutes an auditory agnosia, which may be selective for particular kinds of sounds; aberrant ‘excessive’ processing may manifest as auditory hallucinations. These disorders of auditory cognition commonly coexist.

**Fig. 1:**
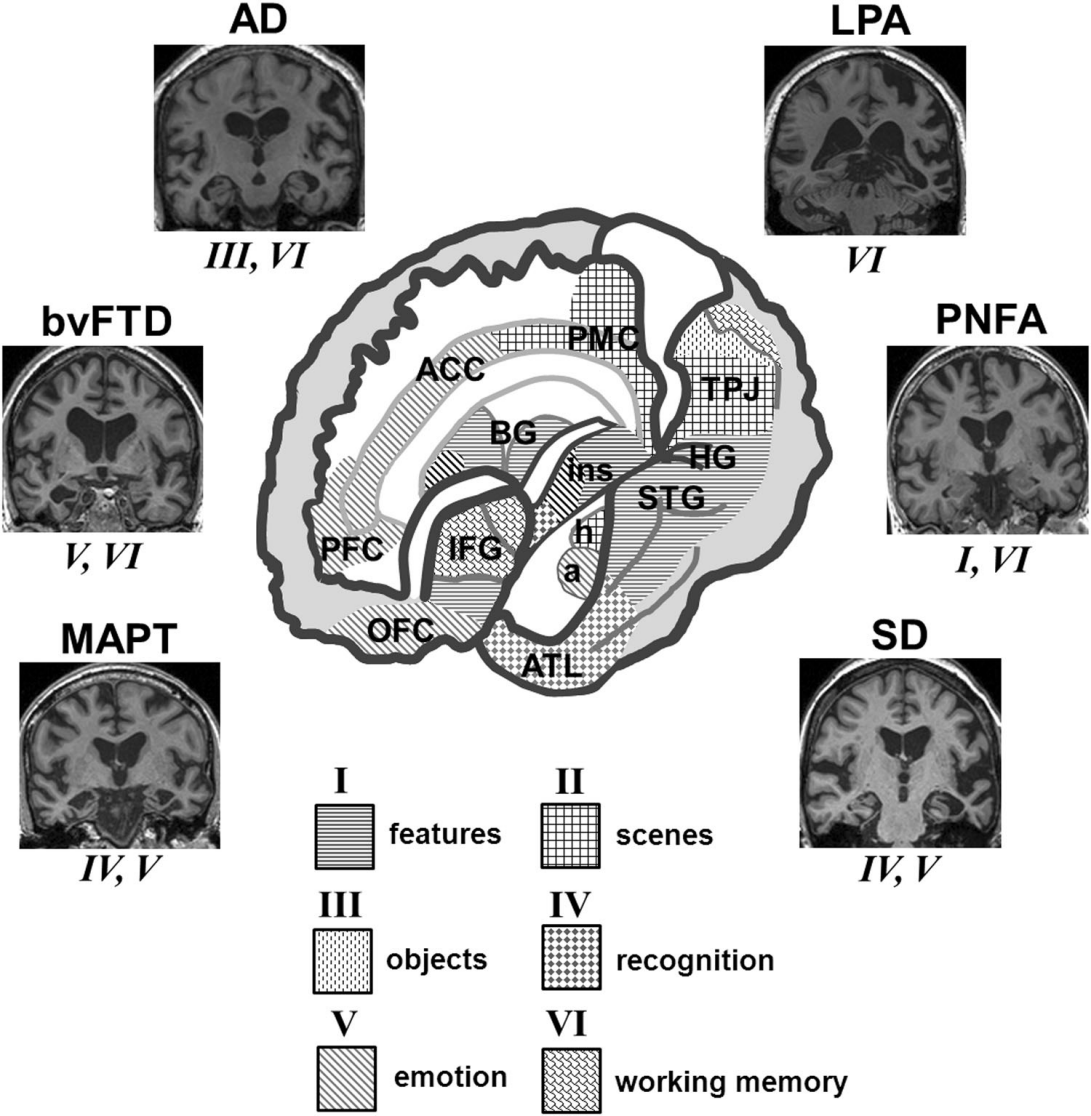
Neuroanatomical signatures of disordered auditory cognition in dementias. The cutaway brain schematic (*centre*) shows cerebral networks that mediate key components of auditory cognition, coded *I* to *VI* (*below*) and based on clinical and normal functional neuroanatomical evidence (see Tables 1, 2); ‘features’ here subsumes acoustic feature detection and analysis, ‘objects’ corresponds to auditory apperceptive processing and ‘recognition’ corresponds to auditory semantic processing. The left cerebral hemisphere is projected forward in the schematic; however, neuroanatomical correlates of auditory cognition are bi-hemispherically distributed, principally, including: *a* amygdala, *ACC* anterior cingulate cortex, *ATL* anterior temporal lobe, *BG* basal ganglia, *h* hippocampus, *HG* Heschl’s gyrus (containing primary auditory cortex), *IFG* inferior frontal gyrus/frontal operculum, *ins* insula, *OFC* orbitofrontal cortex, *PFC* prefrontal cortex, *PMC* posterior medial cortex (posterior cingulate, precuneus), *STG* superior temporal gyrus/superior temporal sulcus/planum temporale, *TPJ* temporo–parietal junction. *Side panels* show characteristic profiles of regional cerebral atrophy (coronal MRI sections) and auditory cognitive functions chiefly affected in selected dementias (see also Table 2): typical Alzheimer’s disease (AD), bilateral symmetrical mesial temporal and parietal lobe atrophy; behavioural variant frontotemporal dementia (bvFTD), asymmetric (predominantly *right-sided*) frontal and temporal lobe atrophy; logopenic aphasia (LPA) variant of Alzheimer’s disease, predominantly *left-sided* temporo-parietal atrophy; microtubule-associated protein tau (MAPT) gene mutations, bilateral (predominantly antero-mesial) temporal lobe atrophy; progressive nonfluent aphasia (PNFA), predominantly *left-sided* peri-Sylvian atrophy; and semantic dementia (SD), asymmetric (predominantly *left-sided*) anterior temporal lobe atrophy

Here, we emphasise the differential diagnosis of auditory symptoms; characterisation of auditory deficits using neuropsychological tests is a complementary enterprise. Together, these approaches define auditory phenotypes, summarised for selected dementias in Table 2; neuroanatomical correlates are shown in Fig. 1.

### Impaired perception of sound features

Patients with dementia may have reduced perception of sound disproportionate to any damage involving cochlea or ascending auditory pathways: this may manifest as cortical deafness (described rarely in prion disease: [43, 44]) or relatively selective ‘word deafness’ or auditory agnosia, more commonly described with progressive nonfluent aphasia and (for unknown reasons) in Japanese patients [45–47]. A useful clinical clue to word deafness is substantially better comprehension of written than spoken language. Speech perception may be particularly vulnerable as it depends on precise temporal feature decoding but may signify a more generic impairment of auditory feature analysis in syndromes with peri-Sylvian degeneration [48, 49].

### Impaired perception of auditory scenes and objects

Frank auditory disorientation is uncommon in dementia though does occur (usually accompanying visual disorientation) in patients with posterior cortical degenerations [50]. However, patients with both posterior cortical atrophy and clinically typical AD commonly report difficulty following conversations and other sounds against background noise, and this may contribute to their avoiding social situations and a general dislike of busy auditory environments [51]. Such symptoms may develop early in the course of the illness and without other evidence of hearing loss and (though often attributed to a nonspecific memory or attentional deficit) may signify AD-associated impairments of auditory scene and auditory spatial analysis, correlated in structural and functional neuroanatomical studies with disintegration of a core parieto-temporal network [6, 9, 10]. Auditory scene analysis depends on accurate parsing of the acoustic stream into constituent sound objects, mediated by sensory computational mechanisms under attentional and executive control; these mechanisms interact in temporo-parietal cortical ‘hub’ regions.

Other symptoms, experienced particularly by patients with AD and progressive nonfluent aphasia suggest auditory apperceptive dysfunction: disproportionate difficulty identifying or understanding sound objects under unusual or degraded listening conditions. The patient may not recognise a familiar voice over a noisy telephone line or may fail to understand a message delivered in an unfamiliar accent [8, 52]). Such symptoms may have a neuroanatomical substrate in posterior peri-Sylvian cortices, similar to that underpinning impaired auditory scene analysis.

### Impaired recognition of sounds

Patients with semantic dementia develop deficits of nonverbal sound recognition (auditory associative agnosia) as part of a pan-modal erosion of semantic memory, linked to antero-mesial temporal lobe dysfunction [48, 53, 54]. Interestingly, in individual cases, there may be relatively preserved knowledge of melodies over environmental sounds [55, 56], perhaps because the abstract, nonreferential meaning systems of music have neural substrates that are separable from those mediating knowledge about the world at large (knowledge of musical instrument timbres may be affected comparably to other categories of objects). Impaired recognition of familiar voices (despite retained ability to distinguish between voices) may be a salient symptom of right temporal lobe degeneration [57, 58]: such ‘associative phonagnosia’ may be relatively selective for voices or accompany other deficits of person knowledge or more generalised auditory agnosia.

### Auditory hallucinations

Patients with semantic dementia commonly report tinnitus (an elementary auditory hallucination), linked to structural alterations in a fronto–temporo–subcortical network [5]; while hallucinations of ‘muffled’ sounds or voices are often reported by patients with Lewy body dementia, frank verbal hallucinations are uncommon and generally occur as a component of more complex, multimodal hallucinations [59]. In contrast, persistent musical hallucinations (typically comprising familiar, banal tunes) are relatively commonly reported in patients with Lewy body disease and less frequently, other dementias [60]: only a minority has significant hearing loss, suggesting that aberrant cortical activity plays a key role though there may be a facilitatory effect from peripheral deafferentation [61].

### Abnormal auditory behaviours

Altered emotional or ‘hedonic’ behavioural responses to sound are increasingly recognised in patients with dementia. Impaired processing of emotional prosody has been described in AD, Parkinson’s disease, behavioural variant frontotemporal dementia, and progressive aphasia syndromes [62–65], and impaired recognition of musical and nonverbal vocal emotions in Parkinson’s disease and frontotemporal dementia syndromes [65, 66]. Many patients with frontotemporal dementia (and some with AD) exhibit sound aversion, while abnormal craving for music (musicophilia) is particularly associated with semantic dementia [51]; these patients may show increased sensitivity to sound (hyperacusis [5]), also described in prion disease [67]. Explicit behavioural responses may dissociate from autonomic responses to sound in dementia syndromes [68, 69]. Altered auditory hedonic behaviours in dementia have been linked to involvement of distributed cortico–subcortical circuitry that processes emotion and reward [51].

## A practical approach to the patient with dementia and altered hearing

**Fig. 2:**
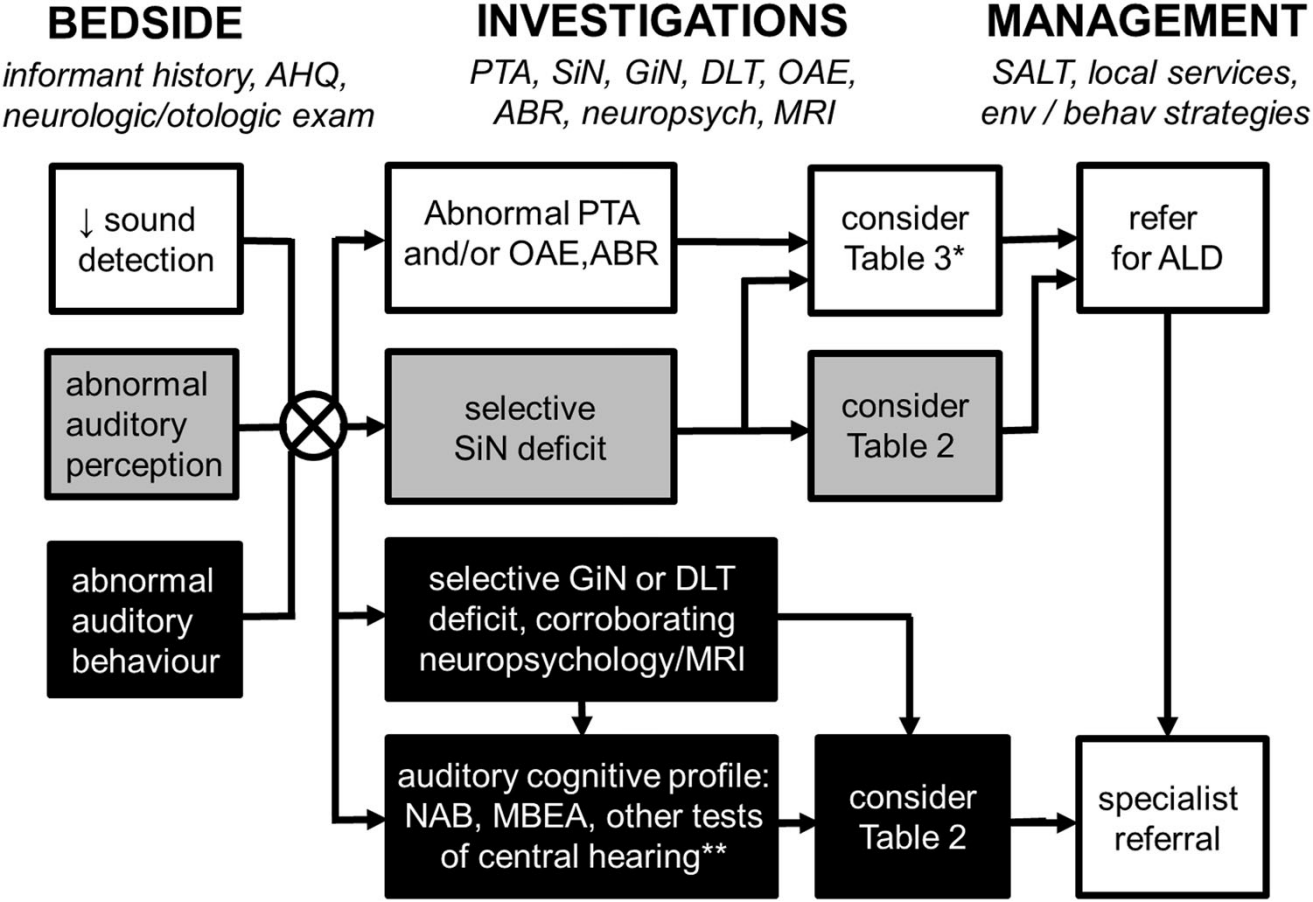
A clinical approach to the patient presenting with cognitive decline and altered hearing. Our approach is based on initial thorough bedside history taking and examination to identify key auditory symptoms (see also Table 4) supplemented by investigations to characterise the nature of the patient’s hearing and cognitive deficits. As clinical symptoms are rarely specific and disorders at different levels of the auditory processing hierarchy frequently coexist, we recommend a core hearing assessment battery in all cases, corroborated by general neuropsychological assessment and brain MRI. Together, these assessments often allow the patient’s hearing deficit to be localised predominantly to the cochlea or ascending auditory pathways (*unfilled oblongs*) or to cerebral circuitry (*black filled oblongs*) and direct further more specific assessment for the diagnoses listed in Tables 2, 3. Other patients will have auditory deficits that are more difficult to localise or may have mixed deficits (*grey oblongs*); speech-in-noise perception is a useful index of real world hearing impairment but needs care in interpretation as this can be affected by pathology at different levels of the auditory system. Management in all cases should involve consideration of environmental and behavioural modification strategies that optimise the patient’s residual hearing function (*see text*) and involvement of multidisciplinary services to assess their needs and plan appropriate care delivery; we have a low threshold for a trial of hearing aids or other assistive listening devices if there is the possibility of a contributing peripheral hearing loss and in patients with more complex or central auditory deficits, onward referral to a specialist cognitive or auditory clinic may be helpful. *Asterisk* particularly in younger patients or where there are associated neurological or systemic features; *double asterisk* more specialised tests of central hearing functions if available may be useful in defining the phenotype of an auditory cortical disorder, particularly where all standard tests of hearing are unremarkable; *ABR* auditory brainstem evoked responses, *AHQ* auditory handicap questionnaire, *ALD* assistive listening device, *behav* behavioural, *env* environmental, *DLT* dichotic listening test, *GiN* gap-in-noise perception, *HA* hearing aid, *MBEA* Montreal Battery for Evaluation of Amusia, *MRI* brain magnetic resonance imaging, *NAB* Newcastle Auditory Battery, *neuropsych* neuropsychology, *OAE* otoacoustic emissions, *PTA* pure tone audiometry, *SiN* speech-in-noise perception

A clinical framework for assessing and managing the patient presenting with cognitive impairment and altered hearing is outlined in Table 4 and Fig. 2.

**Table 4:** Taking the auditory history in patients with cognitive impairment

Domain	Question	Key process probed	Significance
Background	Previous occupation?	Previous cognitive/auditory function, noise exposure	Correct interpretation of hearing tests
	Previous level of musical training and interest, early language development and education?	Prior auditory expertise	Correct interpretation of hearing tests
Course	When was hearing impairment first noticed?	Duration of impairment (relative to cognitive decline)	Nature of underlying disease process
	Has this deteriorated, fluctuated or improved since onset?	Tempo of impairment	Nature of underlying disease process
Symptoms			
Sound detection	Is there a lack of reaction to sounds?	Impaired sound detection	May signify deafness (any cause)
	Is there a tendency to turn up the volume of radio or TV or to ask people to speak louder?	Impaired sound detection	May signify deafness (any cause)
	Is there a complaint that increasing volume makes sounds suddenly seem too loud?	Impaired sound detection	May signify cochlear pathology (‘loudness recruitment’)
Abnormal auditory perception: deficient	Is there particular difficulty following conversations in background noise or over a noisy telephone line?	Auditory scene analysis	May signify a cerebral disorder (e.g., Alzheimer’s disease) in absence of significant hearing loss
	Is there difficulty locating sounds (e.g., an alarm or mobile, a person speaking in same the room)?	Auditory scene analysis	May signify a cerebral disorder in absence of significant hearing loss
	Is there particular difficulty understanding speech versus other sounds?	Feature analysis	May signify word deafness
	Is there more difficulty understanding less familiar accents?	Apperceptive processing	May signify a cerebral disorder in absence of significant hearing loss
	Is there more difficulty understanding a person’s tone of voice (e.g., angry or upset)?	Apperceptive and emotional processing	May signify a frontotemporal dementia in appropriate context
	Has there been any problem recognising familiar voices, music or other sounds?	Semantic processing	May signify auditory agnosia or semantic dementia, in appropriate context
Abnormal auditory perception: excessive	Is there a persistent complaint of buzzing or ringing in the ears?	Tinnitus	May be peripheral or central in origin
	Are other sounds ever heard when no sounds are present?	Formed hallucinations	May signify Lewy body disease, in appropriate context
Abnormal auditory behaviour	Is there intolerance to moderately loud sounds or particular sounds?	Hyperacusis	May signify a frontotemporal dementia in appropriate context
	Has there been any change in liking for or interest in music or other sounds?	Auditory hedonic processing	May signify a frontotemporal dementia in appropriate context

In all cases, a corroborating history should be sought from the patient’s caregiver or other advocate. See also Tables 1, 2, 3; Fig. 2

### Clinical assessment

Hearing function should be assessed in all patients receiving a diagnosis of dementia: to identify a factor that may be detracting from quality of life and impeding care [70], to gauge disability as fully as possible, and to address any reversible peripheral component. Assessment begins with a history to elicit key auditory symptoms (Table 4; Fig. 2) and systematic neurological and otological examination. Hearing impairment can easily go undetected in patients with dementia and may lead to misattribution or overestimation of cognitive compromise [71]; cognitive screening instruments that do not rely on hearing (such as the TYM test [72]) may be preferable to verbally administered tests such as the mini–mental state examination. The patient’s premorbid competence in particular domains (notably, music) should be documented, and an auditory handicap questionnaire may be useful in defining the functional consequences of hearing impairment [73].

In patients with known hearing impairment, any supervening cognitive decline should be thoroughly characterised. This is particularly challenging in those with pre-lingual profound hearing loss. British Sign Language (BSL) users form a close-knit minority group defined culturally as well as linguistically; they often have difficulty in accessing appropriate assessment services and, therefore, may have more advanced dementia when diagnosed. Ideally, assessment of such individuals should be undertaken in a clinic with special expertise in working with deaf people, engaging a neuropsychologist with relevant communication skills and expertise and BSL interpreters trained in the requirements of cognitive assessment. Until recently, there were no normative data on cognition in deaf subjects; however, a cognitive screening instrument based on British Sign Language has now been developed [74]. As with any cognitive assessment, it is essential to obtain a corroborating history from an advocate or caregiver.

### Investigations

Pure tone audiometry and otoacoustic emissions (supplemented by brainstem auditory evoked potentials if available) are relatively simple and well-tolerated techniques to assess cochlear and ascending auditory pathway function in cognitively impaired patients. Measurement of speech-in-noise perception (Table 1) more closely reflects ‘real world’ hearing impairment than pure tone audiometry and (if impaired disproportionately to other indices of cochlear or ascending auditory pathway function) may provide an index of auditory cortical processing; this can be supplemented by tests such as gap-in-noise perception or dichotic listening that depend more sensitively on cortical processing of sound [75] (Table 1). If the patient or caregiver reports symptoms suggesting auditory cognitive dysfunction, a more comprehensive evaluation may be appropriate: age norms are available for the Newcastle Auditory Battery [76] and Montreal Battery for Evaluation of Amusia [77]. General neuropsychological assessment can identify concurrent executive or attentional deficits that may confound performance on auditory tests and may also define associated (visual and verbal) apperceptive or semantic impairments to corroborate any auditory cognitive deficit. If a complex or unusual disorder (such as auditory agnosia) is suspected, referral to a specialist clinic may be indicated. Investigation of any patient with suspected dementia should focus on identifying reversible processes and establishing the primary cause as accurately as possible (for general reviews, see [16–18]); this should include brain MRI, which may further characterise the likely neuroanatomical basis of the auditory deficit (Table 2).

### Management

While effects on specific aspects of cognitive function are difficult to predict, correction of reversible hearing deficits has been shown to benefit global functioning in daily life [20, 21, 23, 78, 79]. Simple interventions such as earwax removal can be highly effective [79]. In addition, prescription of hearing aids and other assistive listening devices where appropriate, environmental modification strategies may be useful in managing altered hearing in patients with dementia. Examples include the use of written communication aids and electronic devices, conducting conversations face-to-face and free of significant background noise, avoiding sounds known to provoke distress, and masking techniques for musical and other auditory hallucinations. Anecdotally, cholinesterase inhibitors may benefit some patients with musical hallucinations [80]; however, there is currently little role for specific pharmacotherapy in managing auditory dysfunction in dementia. Specific auditory training protocols based on speech and nonspeech sounds have been shown to improve speech intelligibility in hearing-impaired adults [81] but have yet to be adequately assessed in dementia. Though music is unquestionably a welcome source of solace for many patients and caregivers and often a useful displacement activity, evidence for a specific role of music therapy in the management of dementia awaits better-controlled trials [11]. Assistive listening devices incorporating a mobile microphone are a promising strategy to compensate for deficits of auditory scene processing [73], but their utility in patients with dementia remains to be established. Management of hearing loss in dementia is an inter-disciplinary enterprise, and close collaboration with audiology, speech and language therapy, and social services is invaluable.

## Conclusions and future directions

Hearing has long been the poor relation of memory and vision in the cognitive clinic. The emerging epidemiological evidence may shortly transform this situation. The comprehensive assessment of hearing in dementia presents both challenges and opportunities. There is a need to develop practical and reliable tests that can disambiguate the effects of peripheral hearing and auditory cognitive dysfunction, to develop auditory interventions directed to cognitively impaired people and to assess these systematically and longitudinally in a range of dementias, referenced to healthy older people. In addition to capturing disability and improving quality of life, a more detailed picture of the spectrum of auditory dysfunction in dementia would have considerable neurobiological and clinical resonance. Sound is a dynamic and computationally demanding sensory signal that engages complex emotional and other behaviours: the processing of sounds taxes brain networks targeted by neurodegenerative pathologies and may yet yield novel cognitive ‘stress tests’ for diagnosis and treatment tracking in these diseases.
